# Fortieth Annual Commencement of the Baltimore College of Dental Surgery

**Published:** 1880-03

**Authors:** 


### EDITORIAL, ETC.

---

*Fortieth Annual Commencement of the Baltimore College of Dental Surgery.*—The fortieth annual commencement of the Baltimore College of Dental Surgery was held on Thursday evening, March 4th, 1880, at the Academy of Music, in the presence of an audience which occupied every part of that immense building. The members of the Faculty, the Graduating Class, and a number of visitors, including eminent dental and medical practitioners from at home and abroad, occupied the stage, which was rendered very attractive by the beautiful floral display in the form of bouquets and other designs, for presentation to the graduates. The music, which was finely rendered in the form of selections, marches and waltzes, was furnished by the Fifth Regiment Orchestra, Prof. Itzel, Director.

The exercises commenced with prayer by the Rev. F. H. Kerfoot, of Eutaw-Place Baptist Church.

The announcement of the graduates was made by Prof. Ferdinand J. S. Gorgas, Dean of the College, together with the authority by which the degree of "Doctor of Dental Surgery" was conferred by the institution. By a singular coincidence the graduating class at this, the *fortieth* annual commencement numbered *forty* members.

The Dean then conferred the degree of "Doctor of Dental Surgery" upon the following gentlemen:

| | | Subjects of Thesis. |
|---|---|---|
| C. James Barber, | *New York.* | —Neuralgia Faciei. |
| Frank A Barrett, | *Dist. Columbia.* | —Alveolar Abscess. |
| John D. Basehore, | *Pennsylvania.* | —Neuralgia. |
| John S. Billopp, | *Maryland.* | —Irregularity of the Teeth. |
| John H. Burnett, | *South Carolina.* | —Sensitive Dentine. |
| Oscar Frederick Coe, | *New York.* | —Pericementitis. |
| Frederick H. Cole, | *New York.* | —Anatomy of the Teeth. |
| Pastor A. Cooke, | *South America.* | —Irritation. |
| H. V. Desportes, | *South Carolina.* | —Treatment of the Dental Pulp. |
| Josiah W. Foreman, | *Virginia.* | —Dental Replacements. |
| James W. Gorden, | *Maryland.* | —Preservation of Dental Organs. |
| Milton H. Gross, | *Pennsylvania.* | —Inflammation. |
| William T. Harban, | *Maryland.* | —Dental Caries. |
| Joseph S. Hartman, | *Virginia.* | —Irregularity of the Teeth. |
| William Hawkins, | *Tennessee.* | —Extraction of Teeth. |
| Garnett L. Hills, | *Dist. Columbia.* | —The Temporary Teeth. |
| Nat. A. Hollinshead, | *Georgia.* | —Dental Irregularities. |
| B. M. R. Hopkinson, | *Maryland.* | —Replacement of Oral Organs. |
| Louis C. F. Hugo, | *Dist. Columbia.* | —Irregularities of the Teeth. |
| Thomas M Hunter, | *North Carolina.* | —Dental Caries. |
| Albert B. King, | *Maryland.* | —Dental Caries. |
| Alexander Leeds, | *Maryland.* | —Diseases of Dental Pulp. |
| Frank P. Lewis, | *Pennsylvania.* | —Stomatitis. |
| Burrows Nelson, | *Dist. Columbia.* | —The Antrum and its Diseases. |
| Carl H. E. Obermuller | *Germany.* | —Fracture of Inferior Maxillary. |
| John C. Oeland, | *South Carolina.* | —Alveolar Abscess. |
| Elisha T. Payne, | *New York.* | —Methods of Practice from [Experience. |
| Samuel A Peden, | *Pennsylvania.* | —Plaster of Paris for Impression. |
| James A. Peirce, | *Virginia.* | —Irregularities of the Teeth. |
| Marion Pirkey, | *Virginia.* | —Preservation of the Teeth. |
| J. M. Quattlebaum, | *South Carolina.* | —Materials for Filling Teeth. |
| Henry L. Rankin, | *Virginia.* | —Digestion. |
| Abraham V. Robbins, | *Pennsylvania.* | —The Brain. |
| J. R. Smith, | *South Carolina.* | —The Mandible. |

| | | |
|---|---|---|
| M. F. Thompson, | *Dist. Columbia.* | —Irregularities of the Teeth. |
| Louis A. Thurber, | *Louisiana.* | —Development of Tooth [Structure. |
| John H. Twyman | *Kentucky.* | —Neuralgia. |
| I. N. Van De Water, | *New York.* | —Operative Dentistry. |
| Louis G. Wietfeldt, | *Germany.* | —Irregularities of the Teeth. |
| John L. Wolf, M. D., | *Dist. Columbia.* | —Operative Dentistry. |

The Honorary Degree was conferred upon Thomas Brian Gunning, of New York.

After the conferring of degrees, Dr. Gorgas introduced Gen. Bradley T. Johnson, who said he had been assigned the pleasant duty of giving the graduates of 1880 a godspeed upon their future course. He could understand that after years of residence in Baltimore a man would form ties which would bind him to her people, for on the other side of the Potomac it was said that when a good Marylander died he always hoped to go to Baltimore. Possibly of all communities in this land, it is one with the largest, most liberal, most catholic spirit. There has never been a time in Baltimore's history when she did not take to her generous heart men of all nations, and when once she has adopted them she makes no difference between them and those "to the manner born." It was with the good wishes of such a people that this class was starting in life. May the careers of its members in after-life prove that they had learned while living here lessons of truth and honor as well as lessons of science—for truth and honor only would bear them prosperously and happily through life. He then spoke of the advance which science has made in the past century towards controlling the forces of nature so as to make them subserve man's comfort and happiness, and besought them, as scientists, to continue in their generation this great work of civilization. Concluding, in the name of the Faculty, he wished them godspeed in a future of prosperity, founded on their own ability and merit.

The class address was made by Burrows Nelson, grandson of John Nelson, of Maryland, formerly Attorney-General of the United States. B. Merrill R. Hopkinson, a member of the graduating class, sung a solo, winning an enthusiastic encore, and displaying great ability as a singer. The exercises were closed with a benediction by Rev. Mr. Kerfoot.

The class officers were as follows :

*President of the Class*—J. Ryerson Smith.

*Vice-President*—Samuel A. Peden.

*Secretary*—J. M. Quattlebaum.

*Treasurer*—J. D. Basehore.

*Executive Committee:* J. S. Billopp, Chairman; Louis C. F. Hugo, Garnett L. Hills, Albert B. King, I. N. Van De Water, J. H. Twyman, T. M. Hunter, C. J. Barber, F. P. Lewis, F. A. Barrett.

